# A Bayesian network approach to feature selection in mass spectrometry data

**DOI:** 10.1186/1471-2105-11-177

**Published:** 2010-04-08

**Authors:** Karl W Kuschner, Dariya I Malyarenko, William E Cooke, Lisa H Cazares, OJ Semmes, Eugene R Tracy

**Affiliations:** 1 Department of Physics, The College of William and Mary, Williamsburg, VA, USA; 2 Center for Biomedical Proteomics, Eastern Virginia Medical School, Norfolk, VA, USA

## Abstract

**Background:**

Time-of-flight mass spectrometry (TOF-MS) has the potential to provide non-invasive, high-throughput screening for cancers and other serious diseases via detection of protein biomarkers in blood or other accessible biologic samples. Unfortunately, this potential has largely been unrealized to date due to the high variability of measurements, uncertainties in the distribution of proteins in a given population, and the difficulty of extracting repeatable diagnostic markers using current statistical tools. With studies consisting of perhaps only dozens of samples, and possibly hundreds of variables, overfitting is a serious complication. To overcome these difficulties, we have developed a Bayesian inductive method which uses model-independent methods of discovering relationships between spectral features. This method appears to efficiently discover network models which not only identify connections between the disease and key features, but also organizes relationships between features--and furthermore creates a stable classifier that categorizes new data at predicted error rates.

**Results:**

The method was applied to artificial data with known feature relationships and typical TOF-MS variability introduced, and was able to recover those relationships nearly perfectly. It was also applied to blood sera data from a 2004 leukemia study, and showed high stability of selected features under cross-validation. Verification of results using withheld data showed excellent predictive power. The method showed improvement over traditional techniques, and naturally incorporated measurement uncertainties. The relationships discovered between features allowed preliminary identification of a protein biomarker which was consistent with other cancer studies and later verified experimentally.

**Conclusions:**

This method appears to avoid overfitting in biologic data and produce stable feature sets in a network model. The network structure provides additional information about the relationships among features that is useful to guide further biochemical analysis. In addition, when used to classify new data, these feature sets are far more consistent than those produced by many traditional techniques.

## Background

The use of mass spectrometry to search for proteins that are indicative of disease has greatly accelerated the "discovery" phase for biomarkers. However, this increase in initial discovery has resulted in very few biomarkers that survive subsequent testing using new data. One of the major causes of the high false positive rate of biomarker candidate discovery is the problem of overfitting classifiers built from small sample sets with many observed variables [1]. A mass spectrum of a biologic sample, such as those obtained via MALDI-TOF profiling of blood serum, may have tens or even hundreds of thousands of time points in the signal. Even after signal processing to reduce noise and increase sensitivity, we still find up to hundreds of time positions that have molecular abundance "peaks" representing the existence of a concentration of some molecule, typically a protein in our application. Our sample sets, unfortunately, consist of perhaps a few hundred samples of various disease states - on the order of the number of features found in the spectra. Much of our group's other work has increased the efficiency of our signal processing [2-4], resulting in ever more features--albeit with more precise abundance measurements--through which we must sort to find those that are diagnostic of a particular disease.

With such small sample sets (compared to the number of features), most typical statistical methods will find many features with correlations to the presence of disease--some likely to be strong, even through random chance. We seek to reduce the number of falsely diagnostic candidate features, and simultaneously determine relationships between features to provide additional information that may help identify proteins for further study. To this end, we have developed a relatively simple method of creating a Bayesian belief network (BN) that starts with the disease state (or class) as the root node, and attempts to organize spectral features that impact knowledge of the disease state. We have chosen model-free criteria to assess relationships, and criteria on which to judge the stability of the resulting network, that is, our confidence that the result will apply to future data, and not lead to a diagnostic "dead end."

After briefly introducing the major mathematical tools necessary to perform the Bayesian network analysis, we will describe our method of building the network structure and testing the stability of the links. We will then describe our application of the method to two sets of mass spectrometry data. The method is first tested on data that was artificially generated, with pre-chosen diagnostic features, specific relationships between features, and large variability carefully introduced to mimic common measurement errors and instrumental variability found in TOF-MS systems [2,5]. The second set of data was derived from a 2004 Institutional Review Board-approved leukemia study conducted at the Eastern Virginia Medical School (EVMS). We will then compare our method's results to several traditional feature selection methods. We will show that the feature set selection using our method is more stable, and, in the case of the leukemia data, better at predicting the error rates achieved when previously withheld (lockbox) data is classified.

### Mathematical Tools

Two primary mathematical tools were needed to implement the BN feature selection method. The Bayesian network itself is a method of encoding the (in)dependencies among random variables. In our application, the variables were the relative intensities of the peak signal at mass positions with significant abundances in at least a subset of sample spectra.

To build the Bayesian network, we borrow from the information theorists a simple, but powerful, model-free test for independence--mutual information. Mutual information is a measure of the information that knowledge of one variable's value (a molecular abundance, say) provides about another (for example, the disease class). For two variables X and Y, the mutual information between them is, in the discrete case,(1)

where x represents all the possible values X can take, similarly with y, and P(x, y) represents the joint probability that X takes on the value x and Y takes on the value y. The mutual information ranges from zero, representing independence between X and Y, to the log of the number of values that X or Y can take. The base of the log is arbitrary, and we use 2 as is conventional in information theory. The maximal value is attained when knowledge of X always provides perfect prediction of the value of Y (or vice versa).

A Bayesian network is, at its most basic level, a formula for a joint probability distribution of a set of variables, such as P(A,B,C,D,...). This formula, which summarizes all the dependencies among the variables, can be represented graphically by use of a directed acyclic graph, or DAG. When the joint probability distribution is determined by the structure of the BN, it can be rewritten in the form P(A|B,C,D,...), or "the probability of A given B and C and D...." When associated with the variables A = "disease class" and B,C,D,... = "data measurements," this represents a classifier. Writing the joint probability distribution from the DAG is straightforward; details can be found in Jensen [6].

**Figure 1:**
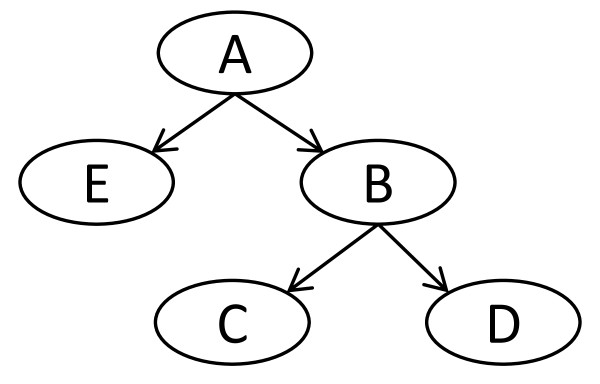
**Example Bayesian network**. A Bayesian network is a directed acyclic graph whose nodes represent random variables. In our work, the root node (A in this figure) always represents the disease state variable, and all other nodes represent the abundance value of specific mass spectrum features. Arcs are assumed to represent causality, so that the state of A causes a change in the probability that B will take on a certain value.

The DAG has two elements: nodes for each variable in the problem, which we will represent as ovals, and arcs, or lines between nodes. The arcs are directed, so that they point (with an arrow) from one node to the other. The graph is acyclic; there are no nodes where it is possible to start, and then return, by following a set of directed arcs (also called a path). Figure 1 represents a simple DAG with five nodes.

The DAG encodes a set of facts about the relationships between the variables in the distribution it models. Arcs represent dependencies, so that, in the most basic case of only two nodes, an arc is drawn if they are dependent and no arc if they are independent. The DAG is simply a way to represent all the dependency information in a particular system of variables; mathematical theorems about various dependencies are then represented as easily visualized operations on the DAG [6].

The general BN has no particular root node, although it will have one or more nodes that have no parents (nodes from which an arc emanates). One aspect of our problem allows us to simplify the intractable problem [7] of exact "structure learning," or assembling, the correct BN for the data. Since we wish to discover features diagnostic of the disease class, we can place the variable representing the class at the root of the BN and work downward.

To do this assumes that the presence of the disease (or more correctly, the diagnosis provided), is causal to the variability in the ion abundance for a particular feature, and that the causality will emerge as a link in the BN. Assumptions of this type underlie much of the work done with belief networks recently, a good discussion can be found in Pearl [8].

An important attribute of the Bayesian network allows a further, and critical, simplification. Connections between variables of the type we seek encode dependencies between the variables, as we have stated. A connection such as A→B→C (with no other arcs) encodes the statement that "although A and C may be dependant, this dependency disappears when B is known." The equivalent joint probability distribution formula encoded by this DAG is P(A,B,C) = P(A) P(B|A) P(C|B).

Thus, when the data is divided up into groups sorted by the possible values of B, we will find that MI(A;C) = 0, and deduce that they are independent. Of course, building the BN goes the other way - such independencies are estimated from observations and built into the resulting DAG.

If we consider B to be not a single variable, but instead the set of all variables whose knowledge isolates A from all other variables C, we have found the "Markov blanket" of A. If we can find a Markov blanket of the class variable, we have built a minimal classifier--knowledge of the variables in set B are sufficient to determine the probability of A, without using any of the variables in C. We have used this concept of the Markov blanket of the disease class to perform feature set selection. We will look further, however, as variables which connect to the Markov blanket might still be of biochemical importance in understanding the disease, even though they are not the primary "biomarkers" in the usual sense.

Another important statistical technique we used is that of *k*-fold cross-validation (CV). We desired to study the stability of the feature selection method, and this type of cross-validation was well suited for that purpose. We repeated the cross-validation a number of times, randomizing the groups each pass. Using that protocol, each case is classified using a different training set. For 10-fold CV, for example, 90% of the cases are used to find classifier parameters to classify the other 10%. By choosing new groups each repetition, a different 90% of cases are used to create the parameters, and a different classification may result.

While "leave one out" cross-validation is intrinsically more stable, it cannot be used in this fashion. Other methods such as bootstrap sampling were considered, but we chose *k*-fold CV (with *k *= 10) for its low bias and variance in this type of problem [9]. To further reduce variance, we used stratified CV, in which a training group is chosen to have approximately the same distribution of classes as the original population.

## Methods

Using these two tools of a Bayesian belief network and mutual information, we have taken the output of our advanced signal processing methods [3,4] and attempted to find diagnostic features, as well as build a classifier that could be used to separate future samples. More detail on the method can be found in Kuschner [10].

The first step in our implementation is to determine all variables that show dependency with the class. We originally attempted to determine a threshold for mutual information significance by repeatedly and randomly permuting the class assignment of our data, computing the mutual information MI(class;feature) for each permutation, and finding the largest "random" mutual information which results. However, due to the mutual information maximization described below, strictly using this baseline mutual information value as a threshold to declare significance resulted in an unrealistic number of features with some connection to the class. The final methodology was empirical, increasing the threshold while monitoring the number of first level features, as well as the fraction of cases misclassified under cross-validation. Feature set sizes uniformly decreased, while error rates had a local minimum, pointing to a stable threshold value. Resource constraints prevented further investigation into a rule-based method of determining a significance threshold, and we hope to improve this aspect of our work at a later date. In the leukemia data described in the next section, a threshold of 3.2 times the "randomized" baseline provided stable feature sets with a reasonable number of features and minimal error rates.

The abundance values for each feature were discretized into 3 bins - high, medium, and low - by maximizing the mutual information of that feature with the class. Thus for each feature, bin boundaries are swept from the maximum to the minimum empirical values, the data is discretized, and MI(class; discretized feature) is found. The bin boundaries that maximize this value are noted and the discrete values of the variables are used for all further calculations.

**Figure 2:**
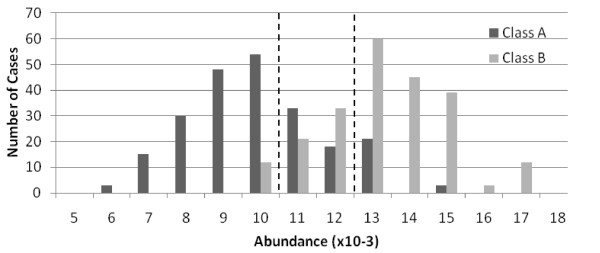
**Three bin discretization**. A histogram of a variable when separated by group shows how central values with little discrimination are isolated, thereby maximizing mutual information between the variable's values and the class.

The three bin method was selected in order to isolate central values which provide little or no discrimination between groups. If a protein has higher abundance in disease samples, for instance, the high bin will have a large difference in the probability of occurrence given disease vs. normal, as will the low bin. The central bin will have nearly the same probability of occurrence regardless of disease state. Diagnostic features will have few cases in the central bin, and the maximized MI will reflect this. Figure 2 illustrates this technique.

With a significance threshold set, all features with MI(class;feature) greater than the threshold are initially considered to have connection to the class variable. Graphically, directed arcs are created in the BN from the class node to each node representing a feature passing this test.

Once a set of features having significant mutual information with the class is established (the "first level features"), they are individually tested against all other features. The MI threshold and discrete values found previously are used (after adjusting for variations in the scale of maximum MI) to determine if connections between features exist. If MI(first-level feature; other feature) exceeds the significance threshold, an undirected arc is placed on the Bayesian network to represent this dependency.

In the case where this test is between a first-level feature and a non-first-level feature, the arc can be directed immediately, given our simplification to the BN based on the causality assumption. However, if this feature-feature link occurs between two first-level features, an additional test is required.

To direct such arcs, we used conditional mutual information. This measurement determines the mutual information *remaining *between two variables when a third variable is known. In practice, the data is partitioned by the third variable's value, and mutual information is measured by(2)

If the connection to the class C is of the form C→V1→V2, then the feature V2 will become independent of the class when the data is partitioned on the values of V1, as discussed previously. In that case, the mutual information MI(C;V2|V1) will drop to zero, indicating the independence and the initial link C→V2 is removed. If this connection accurately reflects the causal situation, however, it will not be true that MI(C;V1|V2) drops to zero--and this link is kept.

This provides a means to organize first-level features with dependencies between them. We compute the conditional MI values and look for MI(C;V2|V1)<<MI(C;V2). If such a drop occurs, we conclude a serial connection C→V1→V2 exists. While we did not find that the conditional mutual information vanished, indicating pure independence, significant drops (>75% of the original value) were often observed. Our results were relatively insensitive to the exact threshold used, but fewer expected connections (like multiply charged states) were organized correctly with this threshold above 90%.

First level feature pairs where such drops were not significant were maintained at the first level, but the arc between them was directed based on the greater of the conditional mutual information results.

These two simple tests resulted in a Markov blanket of features around the class variable and information about correlations between these and other features. The resulting DAG is recorded during each cross-validation trial and is used to classify the test cases. After *k *trials, all cases in the data are classified and an error rate for that trial is recorded. To find that error rate, the probabilistic classification given by the BN (e.g. "probability of disease given this data") is changed to a deterministic classification ("this sample comes from the disease group") using some value, which is 0.5 in all of our results.

By randomizing the list of samples in each of the *k-*fold cross-validation groups *n *times, n*k Bayesian networks are recorded, along with *n *cross-validated error rates. The *stability *of the Bayesian network is examined by noting the *frequency *with which various links appear in this set of results, and the stability of the direction of the arcs between features. An "average" network of most frequent connections can be built, thus enabling the classification of new samples with the most stable connections and parameters found.

The MATLAB code which implements the algorithm described above can be obtained from the MATLAB Central file exchange under the title "WMBAT" http://www.mathworks.com/matlabcentral/fileexchange/24345, or by contacting the authors.

### Data

Two data sets are examined, one artificial and one real. The real data set used is from an Eastern Virginia Medical School (EVMS) study aimed at discriminating between subsets of patients infected with Human T-cell Leukemia Virus type 1 (HTLV-1). Blood sera samples were collected under a protocol developed by the National Institute of Health and EVMS. The samples derived from three major clinical sites and were collected using centralized protocols and kept frozen until processed. The diagnosis and classification of Adult T-cell leukemia (ATL) was made using the World Health Organization classification and Shimoyama criteria. In addition to ATL and healthy individuals the cohort included HTLV-1-infected asymptomatic individuals from the same clinical sites. The acquisition of MS spectra was performed according to protocols described in [11].

EVMS investigators employed an in-house program to assign samples in a randomized matrix pattern to prevent bias between replicates, or clinical status, and chip spot position. All samples were processed in triplicate and the arrayed chips were read in a 48-h period. The matrix codes were assigned by an individual separate from the team that processed the samples so that each phase of the study was blinded with respect to the operator. The code was broken during the classification stage.

Before analysis, the data were divided into two sets. The training set consisted of 145 different patients, of which 78 were classified during the clinical portion as "normal," and 67 with various stages of ATL. After removing spectra from the triplicate processing that had poor signal-to-noise due to instrument problems, we were left with 417 cases for the study. This constituted approximately two-thirds of the data taken; the remaining one-third (with all corresponding replicates) was withheld for validation until a final classifier was created. The data are available at ProteomeCommons.org, under the title "Leukemia'04 TOF spectra parsed into Rdata files."

Signal processing of the spectra was performed using tools created by our group and its collaborators and reported elsewhere [4]. The procedure followed several steps: (1) Removal of an exponentially decaying pedestal (with a time constant of 10000 time steps) presumably created by the low mass matrix products; (2) Peak location and amplitude fitting for each spectra by using a Gaussian line shape with a full-width at half maximum of 11 time steps. This fit weighted each data point's squared error by the inverse of the expected signal to simulate the expected Poisson statistics. Whenever a peak amplitude exceeded 2 times the root-mean-square (RMS) noise in the spectra, that location and amplitude were recorded. (3) All the recorded peaks were aligned across the spectra by shifting the start time of each spectrum to minimize the variation in peak positions found in the spectra. This typically required shifts of as large as ± 6 time steps. (4) A master peak list was generated by including peak locations found in at least 5% of the spectra. (5) Fitted amplitudes were recorded for all spectra at the master peak locations, even those which did not exceed the earlier SNR threshold of 2. (6) The remaining background was removed by smoothing and interpolating each spectrum, after excluding those regions that were within 3 FWMH of a peak or an expected peak. (7) The peak amplitudes were corrected for a systematic decay that was observed in the QC (pooled serum) spectra by increasing each spectrum's amplitude by 0.04% in the order they were taken. This resulted in a net increase of approximately a factor of 2 by the end of the list of spectra. We believe that this was necessary to correct for a laser power decrease over the course of the experimental run. The final result was an array of abundance values for each case at a number of mass-to-charge positions (the peaks). This processing of leukemia data set led to detection of 96 aligned peaks in all 417 spectra in m/z range from 2 to 13 kDa.

The artificial data was created to test the ability of the method to reproduce known relationships. No signal processing is done, but the typical challenges associated with the analysis of real mass spectra are introduced, such as strong correlations between peaks, high variability, convolution of the values of nearby peaks, and the presence of many peaks that are non-diagnostic. Data is generated from Gaussian distributions with means and variances drawn from the leukemia data (which itself has no known underlying distribution). Specific features are chosen to be primary markers, and the mean values of the distributions the two groups are drawn from are one to two standard deviations apart, as was found in the leukemia data. The distribution between groups shown in Figure 2 is from this data set, and simulates the most diagnostic feature found in the leukemia data set. Several other features are chosen to be modifications of the primary features, and random, but bounded, amounts of the primary features are placed in the secondary features. This simulates protein modifications, multiple ionizations, and other systematic events MALDI events [5]. The remaining features are drawn from a single distribution, regardless of class. Other common TOF-MS problems are introduced. For example, one feature has a portion of its values moved to a neighboring feature to replicate signal convolution of nearby peaks. Detail of the creation of the generated data and the MATLAB code used to generate it is at http://kwkusc.people.wm.edu/dissertation/CreateGenData.html.

## Results

The leukemia data set was examined with several traditional feature selection methods, using a classifier as a wrapper, to create a baseline for our method's results. These methods were the naïve Bayesian classifier (NBC), linear and quadratic discriminate analysis (L/QDA), and classification by Mahalanobis distance. The wrapper method of feature selection uses the classifier result to choose which features to include. We used the forward selection method, which adds to the feature set whichever remaining feature best minimizes the error rate of the classifier, and stops when no better feature set can be found.

With this baseline established, the data was processed using the Bayesian network algorithm. Our primary objective was to maximize the stability of feature networks under randomized *k*-fold cross validation, with minimizing error rate a secondary goal.

### Traditional Feature Selection and Classification methods

**Figure 3:**
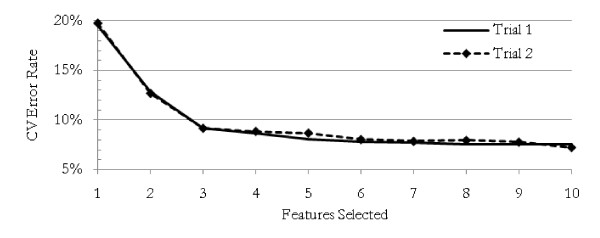
**Error rate as features added**. The cross-validated error rate decreases steadily as features are added according to their ability to increase the accuracy of a naïve Bayesian classifier. Two typical trials are shown. It is possible with a large number of variables to continue to choose ever-larger feature sets with ever-lower error rates, but these larger feature sets are caused by over-fitting and have unstable memberships.

Each of the traditional methods was repeated 30 times using the training data, with feature sets and resulting 10-fold cross-validated error rates recorded for each trial. The best error rate achieved was with QDA. Using an average of just over 9 features, the error rate averaged 3.8%. The results from each method are presented in Table 1. For the NBC, we were able to achieve cross-validated error rates as low as 7% with about 10 features. Figure 3 shows a typical error rate profile as features are systematically added to an NBC feature set.

**Table 1:** Traditional Classifiers Used for Feature Set Selection

Method	Features Selected	Min Error	Lockbox Error
LDA	7.2	7.8 ± 1.2%	21.0 ± 2.6%
QDA	9.7	3.8 ± 0.5%	38.5 ± 1.4%
Mahalanobis	2.8	11.1 ± 0.5%	28.3 ± 2.8%
NBC	6.8	8.4 ± 0.7%	18.9 ± 1.4%

Four different methods of classification are used to select features from the leukemia data set. 30 trials were performed for each. Features Selected represents the average number of features in a feature set when the wrapper determined no better feature set was available. Min Error represents the average 10-fold stratified cross-validated error rate achieved by the final feature set. Lockbox Error is the average error achieved by using the final feature set (and associated parameters) to classify the data which was withheld until the final classifier was built.

One common factor among all these methods was that, with each new trial and subsequent randomization of the group membership for the *k*-fold cross-validation, the features that were selected changed dramatically after the first several choices. Two typical trials of the NBC are shown in Table 2. Additionally, between the various methods, the primary features--those occurring in more than 50% of the trials--were often different. LDA, for example, found features 46, 87, and 96 in 63%, 100%, and 100% of the trials, respectively. Mahalanobis, on the other hand, found only features 95 (100%) and 37 (83%) in more than half the trials.

**Table 2:** Features sets, naïve Bayesian classifier

Set Size	Features Selected	
	***Trial 1***	***Trial 2***
	
**1**	96	96
**2**	87	87
**3**	90	90
**4**	11	32
**5**	54	54
**6**	40	11
**7**	15	6
**8**	46	35
**9**	21	60
**10**	48	53

Two typical trials of a naïve Bayesian classifier wrapper. After the first three features, selection is very unstable. Similar results occurred for all the traditional classifiers.

In this form of feature selection, features that are highly correlated to features already selected are often not subsequently selected. To illustrate, if a new feature is a duplicate of one already chosen, the new feature will show no additional diagnostic power added to the feature set, and will be passed over in favor of another, less diagnostic, feature. We know that feature 95 and 96 are highly correlated, in fact, they represent modified forms of the same protein. In LDA, feature 96 was chosen, but rarely 95. The opposite was true for the Mahalanobis method. Since it is critical to the overall goal of biomarker discovery to understand how each of these modifications is related, this is a near-fatal weakness of these traditional approaches.

Even more critically, the feature sets chosen using the training data *failed to effectively predict *the disease state of the withheld (lockbox) data once it was released. The error rates in classifying the 191 cases from the withheld data far exceeded the cross-validated errors from the training data. QDA, for example, had CV error rates of 3.8+/-0.5%, but was only able to classify the withheld data to about 38% error. All the methods had true errors far outside the predicted range, as shown in Table 1. Such results have been common disappointments in our previous biomarker discovery work.

Trials of the artificial data showed similar results. For example, use of an NBC to select features drove the error rate down to about 1%, but chose many "randomly diagnostic features"--those in which both disease classes were drawn from the same Gaussian distribution and were only selected due to overfitting.

### Bayesian Network Approach

Results using a BN approach with a mutual information score as a filter were more stable. To measure feature selection and error rate stability, a 10-fold stratified cross-validation was run 100 times, with each trial's cross-validation groups randomized.

The method was able to completely recreate the intended network for the artificial data, with two exceptions. One feature that was intended to simulate a multiply-charged ion of a protein modification of a primary diagnostic feature was found connected to the class in a small number of trials. The algorithm was not built to search for such "third-level" features, and the small number of times this feature was connected would have kept it from being added to the final classifier in our methodology. Two features were intended to simulate fragments of a large protein outside the range of measurement (a "hidden variable" in the BN), and one was found frequently connected to the disease class, with the other as its child. The algorithm is not built to detect such hidden variables. One of these features would have been added to the final classifier using our methodology. However, the existence of these fragments in the network may itself lead a researcher to discover the larger, non-measured protein.

All the intended primary diagnostic features were connected to the disease class in 95-100% of trials, showing excellent stability and a nearly perfect discrimination between "real" and "randomly" diagnostic features, unlike the conventional wrapper methods.

**Figure 4:**
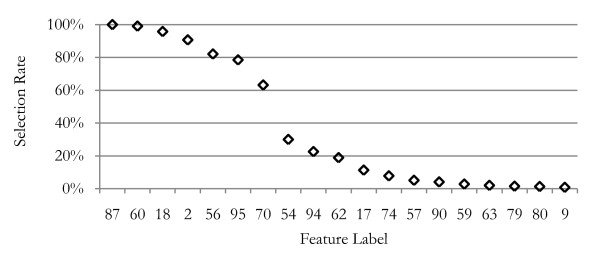
**Feature selection rates**. This graph shows the frequency that features were selected as first level features (connected to the disease class node) during 100 cross-validation trials, resulting in 1000 networks. Seven features have been selected in more than 50% of the trials, and these are used for the final classifier. The 76 features not shown were selected in less than 1% of trials.

Figure 4 shows the frequency of primary connection between features and the disease class for the leukemia data. The feature labels along the horizontal axis of this figure indicate which of the 96 global m/z positions that feature represents. Only the 20 most frequently selected features are shown; beyond that, features were selected less than 1% of the trials. Only 7 features are selected more than one-half of the trials; the 8^th ^most frequently selected feature had a 30% lower selection rate. Only one of these features was selected frequently by the NBC, LDA, and Mahalanobis wrapper methods, two by the QDA method.

**Figure 5:**
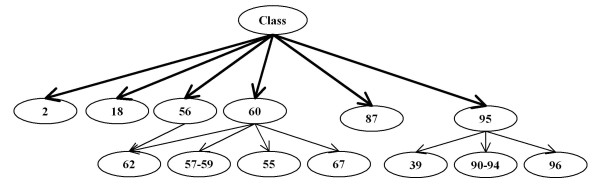
**First level Bayesian network, leukemia data**. This diagram shows the most-likely network as derived from the frequency and stability of the network nodes found during cross-validation. All features show high mutual information with the class variable. The first level features are those which cannot be made independent of the class when conditioned on other features. Features placed on the second (lowest) level below the class have little mutual information with the class when conditioned on the parent feature.

Using a nominal 50% threshold, we can immediately build the first level of the BN below the root node, which represents the disease state. Figure 5 shows the initial BN as thick solid arcs connected to the disease class node, "C." It is important to note that more features may have initially passed the significance test and been placed on the first level of the BN, but subsequent tests showed them to be children of these 7 remaining features.

**Figure 6:**
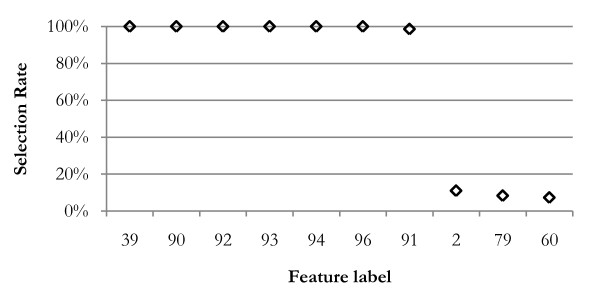
**Features connected to feature 95**. Seven features are frequently found to be children of feature 95 (11,696 Daltons). Feature 39 is a doubly charged ion; others are likely various modifications of the primary molecule. This knowledge allows the researcher to narrow the search to proteins that have the mass of the parent and have modifications that match the masses of the child molecules.

In addition to the primary feature set, the method allows discovery of relationships between primary features and all other features. The thin arcs in Figure 5 show additional relationships found by testing mutual information between features, and organized by testing for conditional mutual information with the disease class. Figure 6 shows the selection rate of the 10 features most frequently selected as children of feature 95, a first level feature. In previous leukemia data study, Semmes et al. [11] had found the cluster containing this feature (among 12) with very low p-values as a discriminator of disease state. Table 3 lists the M/Z values for the features found in Figure 5.

**Table 3:** M/Z values for key features in leukemia data

Feature Number	M/Z value
2	2799
18	3898
39	5879
55	7579
56	7651
57	7780
58	7821
59	7846
60	7864
62	7986
67	8322
70	8455
87	10547
90	11404
91	11486
92	11507
93	11539
94	11641
95	11696
96	11742

96 features were found in the range of mass-to-charge values we used for this. The list of features that were found to be important and the corresponding m/z values are given in this table.

For this data set, families of features were discovered that allowed a preliminary identification of a primary feature based not only on its mass, but on the mass values of related features. Feature 39, for example, has a mass-to-charge ratio almost exactly 1/2 of that of feature 96, indicating that it is the same molecule in a doubly ionized state.

Other features such as 94 and 96 have masses that differ from feature 95 by values that may indicate, for example, loss of an arginine residue. Investigating each of these "children" allows more preliminary information about the "parent" ion and may lead to a more rapid chemical identification. This information is not readily obtainable by a more traditional feature selection method. In fact, we noticed consistent literature reports [12] for a protein (at 11.696 kDa) with this family of modifications, and further experimental testing validated that identification and assignment to serum amyloid A. Thus, the combination of better data processing and analysis improved biomarker ID capabilities [11,12]

As a final test of the technique, an approximate Bayesian network (Figure 5) was created for this data set, using the most frequent results. The entire training set was discretized and probability parameters for the network were estimated empirically from the result. With the final classifier parameters "frozen," the lockbox of previously withheld data was opened, and the spectra pre-processed using the same techniques and parameters as the training data, including using the same global peak list as features.

**Figure 7:**
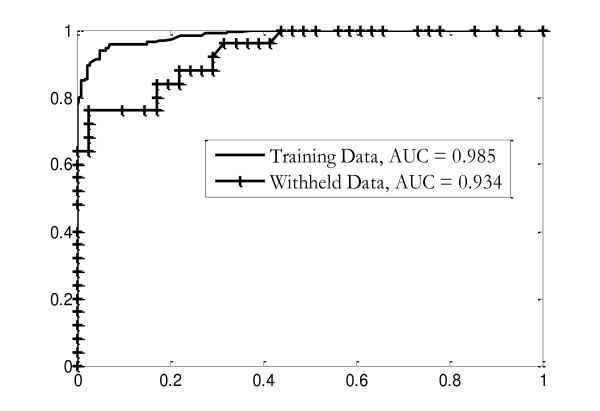
**ROC curves**. ROC curves of the final classifier, which is constructed using the most frequent and stable results from the cross-validation trials. Parameters for the classifier have been learned from the complete training data set; the resulting classifier has been used to reclassify the training data (solid line) and a set of withheld data. The results from the training data are better than the average cross-validated results since the bin boundaries and probability parameters used in the final classifier come from the entire training data set, not a cross-validation subset.

The resulting list of observed variable values for each case was discretized using the boundaries determined from the training data and then classified using the final classifier. Probabilistic results were converted to deterministic using 0.5 as a threshold, as was done for the training set. The results were recorded and then assessed against medical diagnoses recorded in the database. The error rate for this classifier was 15.2%, within the 13.7 ± 1.9% predicted by the cross-validation of the training data. ROC curves for the classification of both the original training set, and the withheld data, are shown in Figure 7. The same feature lists, discretization, and probability tables were used for both sets of data.

The ability of the final classifier to classify the withheld data at a rate similar to the cross-validated error rates for the training data was an important improvement from the poor classification results of the conventional feature selection and classification methods.

## Discussion

The problems expected of more conventional feature selection methods were found in the leukemia data set when the classifier-wrapper methods were applied. Correlated feature groups (such as 90-96) had one feature selected, while the others in the group were overlooked. Subsequent feature selection was unstable due to the high dimensionality of the data and small sample size. This problem is well documented, but often ignored [13].

More problematically, resulting classifiers produced good results on the data they were derived from, but poor results when applied to new data. Given the resources required to validate biomarker candidates, this approach is an inadequate technique for biomarker discovery.

The Bayesian network/mutual information approach provided a much clearer partition between stable and unstable features. In the experiment using artificial data, the BN clearly identified all parent features. It also correctly showed the relationship of nearly all correlated features, including those that were designed to replicate MS-specific effects such as the convolution of peak shapes separated by less than the shape width.

Most promisingly, error rates predicted by many trials of cross-validation were found to agree well with the results of the training data derived BN classifier used on withheld data. This was not true of the traditional methods. The NBC, for example, was not useful for finding diagnostic information past two or three features, even thought the error rate could be driven artificially low by overfitting.

## Conclusions

The process of chemically validating the selected features in the search for biomarkers is costly and time-consuming. Feature selection methods that prevent false positive results are critical to making progress in this field. Feature selection methods using traditional classifiers as wrappers suffer from overfitting in small sample sets, and mishandle information about highly correlated variables. The Bayesian network approach, combined with model-free mutual information scoring, appears to highlight stable features, as well as provide the opportunity to examine relationships between diagnostic features that may assist in identification.

## Authors' contributions

KWK applied the Bayesian network framework, created the analysis software tools, and analyzed the data. ERT provided the theoretical basis for the selection of mutual information as a scoring technique and other statistical methods. OJS led the team that developed the experimental clinical MS protocols, and LHC produced the raw spectra from the blood sera samples. DIM and WEC provided the code for the signal processing and creation of the peak list, and interpreted the results of the feature selection. All authors have read and approved the final manuscript.

## References

[B1] Ioannidis JP Genetic Associations: False or True? Trends Mol Med 9 135 138 10.1016/S1471-4914(03)00030-3 12727138

[B2] Malyarenko D Enhancement of Sensitivity and Resolution of Surface-Enhanced Laser Desorption/Ionization Time-of-Flight Mass Spectrometric Records for Serum Peptides Using Time-Series Analysis Techniques Clin Chem 2005 51 65 74 10.1373/clinchem.2004.037283 15550476 PMC4507422

[B3] Malyarenko DI Resampling and Deconvolution of Linear Time-of-Flight Records for Enhanced Protein Profiling Rapid Commun Mass Spec 2006 20 1 678 10.1002/rcm.2254 PMC7432531 16637003

[B4] Tracy MB Precision Enhancement of MALDI-TOF-MS Using High Resolution Peak Detection and Label-Free Alignment Proteomics 2008 8 8 1530 1538 10.1002/pmic.200701146 18340636 PMC2413415

[B5] Cotter RJ Time-of-Flight Mass Spectrometry 1997 ACS: Washington, DC 326

[B6] Jensen FV Nielson TD Bayesian Networks and Decision Graphs 2007 New York, Springer

[B7] Needham CJ Bradford JR Bulpitt AJ Westhead DR A primer on learning in Bayesian networks for computational biology PLoS Comput Biol 2007 3 1409 1416 10.1371/journal.pcbi.0030129 PMC1963499 17784779

[B8] Pearl Judea Causality 2000 Cambridge, Cambridge University Press

[B9] Kohavi R Mellish CS A study of cross-validation and bootstrap for accuracy estimation and model selection Proceedings IJCAI-95 1995 Los Altos, CA, Morgan Kaufmann 1137 1143

[B10] Kuschner K A Bayesian Network Approach to Feature Selection in Mass Spectrometry Data PhD Dissertation 2009 http://kwkusc.people.wm.edu/dissertation/Kuschner%20Dissertation.pdf 10.1186/1471-2105-11-177 PMC3098056 20377906

[B11] Semmes OJ Discrete serum protein signatures discriminate between human retrovirus-associated hematologic and neurologic disease Leukemia 2005 19 1229 1238 10.1038/sj.leu.2403781 15889159

[B12] Hortin GL The MALDI-TOF Mass Spectrometric View of the Plasma Proteome and Peptidome Clin Chem 2006 52 7 1223 1237 10.1373/clinchem.2006.069252 16644871

[B13] Michiels S Prediction of cancer outcome with microarrays: a multiple random validation strategy The Lancet 2005 365 488 492 10.1016/S0140-6736(05)17866-0 15705458

